# Correction: Soleus muscle weakness in cerebral palsy: Muscle architecture revealed with Diffusion Tensor Imaging

**DOI:** 10.1371/journal.pone.0234582

**Published:** 2020-06-05

**Authors:** Annika S. Sahrmann, Ngaire Susan Stott, Thor F. Besier, Justin W. Fernandez, Geoffrey G. Handsfield

Fig 3 is incorrect. The y-axis ranges 0, 10, 20, 30. The y-axis should range 0, 1.0, 2.0, 3.0. The authors have provided a corrected version here.

**Fig 3:**
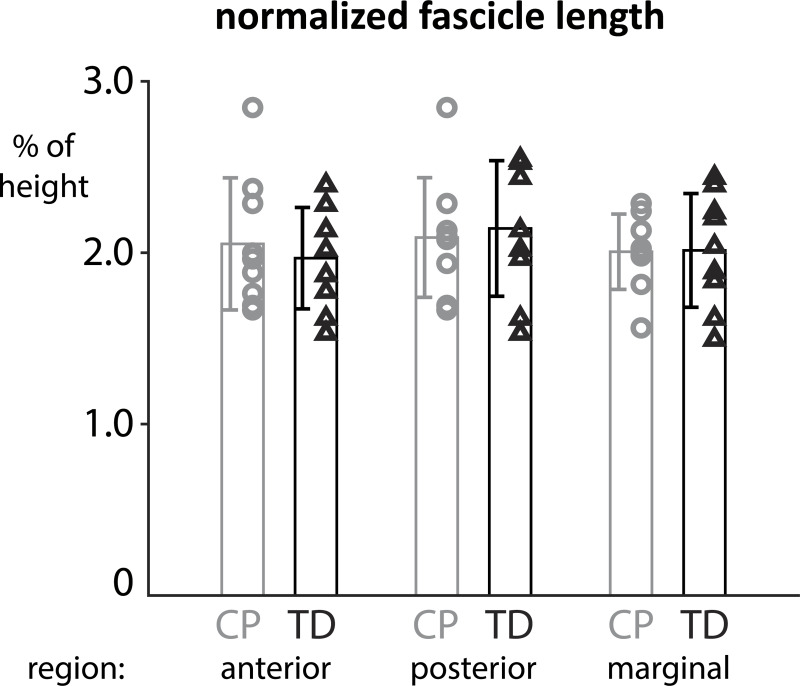
Normalized fascicle lengths in the three regions of the soleus show heterogeneities within and between groups but are not significantly different between CP and TD cohorts.
